# Small Noncoding RNAs in Knee Osteoarthritis: The Role of MicroRNAs and tRNA-Derived Fragments

**DOI:** 10.3390/ijms22115711

**Published:** 2021-05-27

**Authors:** Julian Zacharjasz, Anna M. Mleczko, Paweł Bąkowski, Tomasz Piontek, Kamilla Bąkowska-Żywicka

**Affiliations:** 1 Institute of Bioorganic Chemistry, Polish Academy of Sciences, 61-704 Poznan, Poland; jzacharjasz@ibch.poznan.pl; 2 Center for Advanced Technology, Adam Mickiewicz University in Poznań, 61-614 Poznan, Poland; anna.mleczko@amu.edu.pl; 3 Department of Orthopedic Surgery, Rehasport Clinic, 60-201 Poznan, Poland; pawel.bakowski@rehasport.pl (P.B.); tomasz.piontek@rehasport.pl (T.P.); 4 Department of Spine Disorders and Pediatric Orthopedics, University of Medical Sciences Poznan, 61-854 Poznan, Poland

**Keywords:** small RNAs, miRNAs, tRNA-derived fragments, knee osteoarthritis

## Abstract

Knee osteoarthritis (OA) is a degenerative knee joint disease that results from the breakdown of joint cartilage and underlying bone, affecting about 3.3% of the world’s population. As OA is a multifactorial disease, the underlying pathological process is closely associated with genetic changes in articular cartilage and bone. Many studies have focused on the role of small noncoding RNAs in OA and identified numbers of microRNAs that play important roles in regulating bone and cartilage homeostasis. The connection between other types of small noncoding RNAs, especially tRNA-derived fragments and knee osteoarthritis is still elusive. The observation that there is limited information about small RNAs different than miRNAs in knee OA was very surprising to us, especially given the fact that tRNA fragments are known to participate in a plethora of human diseases and a portion of them are even more abundant than miRNAs. Inspired by these findings, in this review we have summarized the possible involvement of microRNAs and tRNA-derived fragments in the pathology of knee osteoarthritis.

## 1. Introduction

Osteoarthritis (OA) is a highly prevalent musculoskeletal disorder, that affected 303 million people globally in 2017 [1]. It can affect any joint, but preferentially affects the knee, hip, spine and upper limb joints. OA has a considerable impact on the individual patient, resulting in pain and disability, and on society. Furthermore, the economic burden of OA on patients and society is considerable. In 2016 this large disease burden led to the submission by Osteoarthritis Research Society International (OARSI) of a white paper, describing osteoarthritis as a serious disease [2].

The prevalence of OA is increasing due to the ageing population and an increase in related factors such as obesity. According to the United Nations, by 2050, people aged over 60 will account for more than 20% of the world’s population [3]. Of that 20%, a conservative estimate of 15% will have symptomatic OA and one-third of these people will be severely disabled. This means that by 2050, 130 million people will suffer from OA worldwide, of whom 40 million will be severely disabled by the disease [4]. Expenditures associated with OA include costs for adaptive aids and devices, medicines, surgery and time off at work [5].

As OA is a multifactorial disease, the underlying pathological process is closely associated with alterations of various transcripts [6,7,8,9,10], including noncoding RNA species (ncRNAs), especially small ncRNAs, which are <200 nucleotides (nt) in length. It has been reported that unique small ncRNA expression signatures are believed not only to characterize specific types of cells, but also to be indicative of particular diseases [11]. Thus, small ncRNAs can not only reveal key insights into transcriptional mechanisms of diseases, but they can also serve as diagnostic markers.

**Figure 1:**
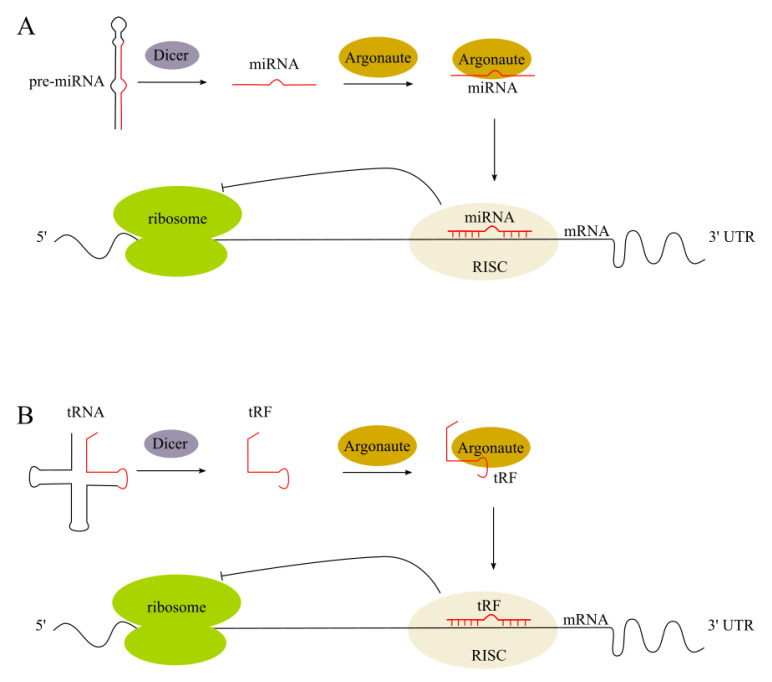
The classic endogenous miRNA pathway and miRNA-like tRF function. (**A**) Pre-miRNA is cleaved by the Dicer enzyme into the miRNA duplex. The guide strand is uploaded onto the RNA-induced silencing complex (RISC) to regulate gene expression by causing either target mRNA degradation or translation repression; (**B**) mature tRNA is cleaved by the Dicer enzyme into the tRF, which is uploaded onto the RISC to regulate gene expression.

There are key small ncRNA types with a size range of 14–35 nt that are highly important for diagnostic biomarker discovery and the development of therapeutic agents [12,13,14,15]. These include microRNAs (miRNAs) and tRNA-derived fragments (tRFs). In human tissues, both types of these small ncRNA molecules are abundant, with expression patterns that depend on a person’s sex and population origin. The size similarity between most tRFs and miRNAs led researchers to hypothesize that tRFs behave like miRNAs and influence protein abundance in an Argonaute-dependent manner. Several studies have indeed showed the association of a number of tRFs with Argonaute proteins (summarized in [15]) and miRNA-like function (Figure 1).

Multiple in vitro and in vivo studies have reported miRNA involvement in OA onset and progression, by targeting cartilage-associated genes. The connection between other types of small noncoding RNAs, especially tRNA-derived fragments and knee osteoarthritis is still elusive. There are only limited reports so far describing the potential interplay between knee OA and small ncRNAs other than miRNAs. Thus, there is an urgent necessity to deepen the knowledge about the small noncoding RNAs in knee osteoarthritis development and progression. This review article aims to provide a comprehensive update on the evidence for two types of small ncRNA roles in OA: microRNAs and tRNA-derived fragments.

## 2. Pathophysiology and Diagnosis of Knee Osteoarthritis

The knee is the largest synovial joint in humans, it is composed by osseous structures (distal femur, proximal tibia and patella), cartilage (meniscus and hyaline cartilage), ligaments and a synovial membrane. The latter is in charge of the production of the synovial fluid, which provides lubrication and nutrients to the avascular cartilage [16]. Given the high use and stress of this joint, it is a frequent site for painful conditions, including knee osteoarthritis [17].

Osteoarthritis is classified into two groups according to its etiology: primary (idiopathic or non-traumatic) and secondary (usually due to trauma or mechanical misalignment). The severity of the disease can be graded according to the radiographical findings by the Kellgren–Lawrence (KL) system described in 1957 [18]. It was believed that knee OA was exclusively a degenerative disease of the cartilage, however, the latest evidence has proven that OA is a multifactorial entity, involving multiple causative factors, such as: trauma, mechanical forces, inflammation, biochemical reactions and metabolic derangements [19]. It is also known that the cartilaginous tissue is not the only one involved. Given its lack of vasculature and innervation, the cartilage by itself is not capable of producing inflammation or pain, at least in early stages of the disease. Hence, the source of pain is mainly derived from changes to the non-cartilaginous components of the joint, like the joint capsule, synovium, subchondral bone, ligaments and peri-articular muscles [16,19]. As the disease advances, these structures are affected and changes can become evident [20].

Osteoarthritis is diagnosed clinically. Common clinical symptoms include: knee pain that is gradual in onset and worse with activity, knee stiffness and swelling and pain after prolonged sitting or resting.

The diagnosis of knee OA continues to rely heavily on X-rays and is based upon a combination of characteristic structural features and pain symptoms [21]. The typical changes seen on X-ray include: joint space narrowing, subchondral sclerosis (increased bone formation around the joint), subchondral cyst formation and the presence of osteophytes (the most specific radiographic marker for OA, although it is indicative of relatively advanced disease and limb malalignment).

Although conventional X-rays are the most inexpensive and readily accessible method of imaging to confirm a diagnosis of knee OA, magnetic resonance imaging (MRI) is becoming an increasingly important diagnostic tool. Compared to conventional X-rays which only can show bony changes, MRI has the benefit of providing information about cartilage, periarticular structures (tendons), and can show edema in the subchondral bone.

Notwithstanding, both, X-ray and MRI features are of limited value in the early, mildly symptomatic stages of knee OA.

## 3. Biochemistry of Osteoarthritis

**Figure 2:**
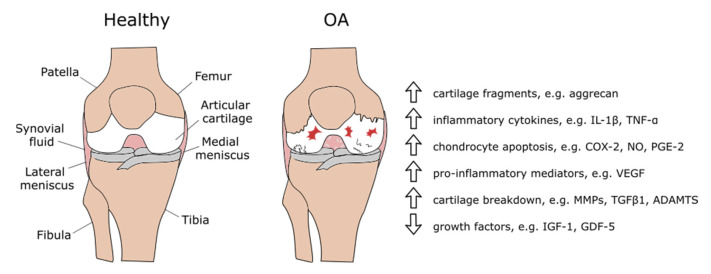
Biochemical changes in knee osteoarthritis.

To understand the regulatory function of small ncRNAs on particular gene expression in knee osteoarthritis, first it is important to present an outline of the biochemical changes during OA (Figure 2). Articular chondrocytes constitute about 1–5% of the total cartilage volume [22]. They produce and maintain the cartilaginous matrix, which consists mainly of collagen and proteoglycans. Chondrocytes provide a balance between anabolic and catabolic activities that protect the aggrecan structure.

Osteoarthritis initiates with an increased turnover of chondrocytes [23]. Apoptosis, autophagy and necroptosis play crucial roles in the chondrocyte death. Such abnormal death of chondrocytes not only reduces their number, but also initiates the degeneration of the cartilage [24,25]. Deprivation of the cartilaginous matrix results in an imbalance between the cartilage synthesis (anabolic) and resorption (catabolic) processes in the joint (as reviewed in [26]). In the early stages of OA, increased synthesis of the extracellular matrix (ECM) components is exceeded by their degradation, due to increased synthesis and activity of proteases. The expression of pro-catabolic target genes is mediated by the nuclear factor kappa-light-chain-enhancer of activated B cells (NF-κB), MAPK (mitogen-activated protein kinases) and SMAD signaling, whereas pro-inflammatory targets are activated downstream of MAPK and NF-κB signaling. TGF-β (transforming growth factor beta) signaling is globally downregulated in OA, with especially negative regulation of the anabolic SMAD3 mediated pathway.

Mechanical strain causes the upregulation of the cytokines. The main cytokines that cause the degradation in the synovia are the interleukins: IL-1β, IL-6, IL-17 and tumor necrosis factor-alpha (TNF-α) [27,28]. 

IL-1β is one of the most important proinflammatory cytokines. It is a suppressor of type II collagen (Col2A1) and aggrecan synthesis, which are key constituents of the cartilage [29,30]. Furthermore, IL-1β induces the production of a number of cytokines and chemokines which contribute to the inflammation. Mouse models have shown that IL-1β plays important roles in pain sensitivity [31].

IL-6 is a 184 amino acid residue protein, which plays a pro-inflammatory role in the pathophysiology of OA. Healthy chondrocytes produce low amounts of IL-6 without the presence of a stimulating agent, but when exposed to IL-1β, TNF-α or interferon-γ, the chondrocytes increase IL-6 expression [32,33]. IL-6 inhibits the production of Col2A1 [34].

TNF-α is a 17 kDa protein produced predominately by activated macrophages which affect the production of other cytokines. The upregulation of TNF-α correlates with pain, joint stiffness and higher radiographic severity of OA [35,36].

The upregulation of cytokines causes the induction of proteolytic matrix metalloproteinases (MMPs) and a disintegrin and metalloproteinase with thrombospondin motifs (ADAMTS), which enzymatically disrupt the cartilage structure. MMPs are a family of zinc-dependent proteolytic enzymes responsible for the cleavage of a variety of ECM proteins [37]. Most MMPs, including MMP-1, MMP-2, MMP-3, MMP-8, MMP-9, MMP-10, MMP-14 and especially MMP-13, are involved in the turnover of ECM and the associated destruction of articular cartilage in OA [38]. MMP-13 causes a rapid breakdown of type II collagen, but also targets other matrix molecules such as type I, III, IV, IX, X collagen, perlecan, osteonectin and proteoglycan and it is likely involved in matrix turnover in healthy cartilage [38]. The ADAMTSs are a family of 19 secreted metalloproteinases. (ADAMTS)-4 and -5, degrade aggrecan, a primary structural component of the cartilage ECM, except of collagen [39].

The destruction of type II collagen promotes the hypertrophy of the chondrocytes through the bone morphogenetic protein (BMP) pathway, thus exacerbating the progression of the osteoarthritis [40]. 

Other elements and side products which further increase cartilage degradation and play a role in osteoarthritis include insulin-like growth factor 1 (IGF-1), and chondrodegradative enzymes [35]. The conversion of arachidonic acid through cyclooxygenase (COX-2) leads to the production of prostaglandins, which are physiologically important mediators in tissue repair and prostacyclins [41].

## 4. Transcriptomics of Knee Osteoarthritis

The completion of the Human Genome Project has revealed that protein-coding genes comprise only about 1.5% of the human genome and that the majority of the genome is transcribed and produces a wide spectrum of noncoding RNA species. Therefore, the connection between ncRNAs and OA development is the focus of an increasing number of studies.

Noncoding RNAs can be classified according to their size [42]:

- small noncoding RNAs are <200 nucleotides (nt) in length and include small interfering RNAs (siRNAs), piwi-interacting RNAs (piRNAs), microRNAs (miRNAs), transfer RNAs (tRNAs), tRNA-derived fragments (tRFs), small nuclear RNAs (snRNAs) or small nucleolar RNAs (snoRNAs), snoRNA-derived small RNAs (sdRNAs) and small Cajal body-specific RNAs (scaRNAs)
- long noncoding RNAs are longer than 200 nt and may comprise thousands of nucleotides, like ribosomal RNAs (rRNA).

Noncoding RNAs fulfill relatively generic functions in cells, such as rRNAs and tRNAs involved in mRNA translation to proteins, snRNAs involved in splicing or snoRNAs involved in the modification of RNAs [42]. Moreover, a whole spectrum of small ncRNAs regulate gene expression at different levels, using different mechanisms. Therefore, the unique small ncRNA expression signatures are believed to not only characterize specific types of cells, but also to be indicative of particular diseases [11,43,44,45,46,47,48,49]. Thus, small ncRNAs can not only reveal key insights into transcriptional mechanisms of diseases, but they can also serve as diagnostic and prognostic markers.

### 4.1. MicroRNAs in Knee OA

MicroRNAs are noncoding molecules of about 19–23 nt that are derived from stem-loop precursors (pri-miRNAs and pre-miRNAs) in animals and plants [50]. These pri-miRNA molecules undergo maturation in nucleus via Drosha/DGCR8 processing into pre-miRNA [51,52]. Pre-miRNA, ~70 nt long hairpin structured intermediate molecules [53], are exported to the cytoplasm from the nucleus via Exportin-5 [54], followed by the RISC loading complex incorporation of pre-miRNA. Within this complex, Dicer cleavage of pre-miRNA occurs, resulting in the formation of miRNA/miRNA* (guide strand/passenger strand) complex [55]. This duplex is further processed in Argonaute-protein-members-dependent manner, giving rise to the mature (guide strand) miRNA molecule.

The main role of miRNA in the human body is post-transcriptional gene expression regulation by mediating the degradation of mRNA and also regulating translation through canonical and non-canonical mechanisms. miRNA mediate mRNA interference by sequence-specific binding to the 3’UTR (canonical) or 5’UTR (non-canonical) regions [56,57]. MicroRNAs regulate more than 50% of protein coding mRNAs in mammalian cells [58]. To date, 2588 human mature miRNAs have been identified and are currently included in miRBase [59].

Deregulation of miRNA levels might lead to multiple diseases, e.g., many types of cancer [60,61,62], cardiovascular diseases [43], sepsis [63] or nervous system [45,49] disorders. In knee osteoarthritis, alterations in the levels of miRNAs in cartilage may result in an aberrant expression of the target genes, thereby disrupting the cartilage homeostasis. Multiple in vitro and in vivo studies have reported miRNA involvement in OA onset and progression, by targeting cartilage-associated genes [64]. It is now known that miRNAs regulate the expression of genes involved in pathways altered in OA chondrocytes, such as apoptosis [65], expression levels of MMPs and ADAMTS [66] and chondrocyte signaling [67]. To date, it has been reported that about 80 miRNAs are involved in the pathology of OA and many excellent reviews have already presented miRNA function in OA, e.g., [68,69,70]. However, the progress in novel high-throughput technologies of small ncRNA identification is so fast, that we have noticed the need for updating and summarizing the consequences of deregulation of miRNA expression levels during knee osteoarthritis. Here we present an updated summary of miRNAs which are differentially expressed in OA (Table 1).

Multiple miRNAs have been found to be overexpressed in OA, including miR-146a, miR-181, miR-16, miR-21, miR-30 or miR-365. One of the most studied miRNAs is miR-146a [81,82,83]. Other miRNAs were also reported to be significantly correlated with the pathogenesis of OA. The levels of 380 plasma miRNAs in patients with OA and healthy controls were compared. The results indicated there were 12 overexpressed detectable miRNAs, including miR-16, miR-20b, miR-29c, miR-30b, miR-93, miR-126, miR-146a, miR-184, miR-186, miR-195, miR-345 and miR-885-5p, that were altered in the OA and could be released into the plasma [71]. Furthermore, six miRNAs, including miR-23a-3p, miR-24-3p, miR-27b-3p, miR-29c-3p, miR-34a-5p and miR-186- 5p, were significantly upregulated in late-stage OA synovial fluid compared with early-stage OA synovial fluid [74]. Among them, miR-29c and miR-186 have been reported to be overexpressed in the plasma of early-stage OA synovial fluid compared with healthy controls.

The downregulation of miRNAs, including miR-132, miR-25, miR-28, miR-140, miR-191, miR-342, miR-454, miR-let-7b, miR-let-7a, miR-27a, miR-329, miR-708, miR-934, miR-877, miR-1180, miR-320b and miR-663a, has been identified in the progression of OA. Downregulation of miR-140 during OA has been widely studied [97,98,99,100,101]. Its expression was decreased in osteoarthritic cartilage compared with healthy cartilage. IGFR was identified as the primary target of miR-140, which is involved in the regulation of metabolic processes contributing to the pathophysiology of OA [71]. Additionally, ADAMTS5, MMP-13, IGFBP5 and RALA, which play important roles in mediating the degradation of cartilage matrix, modulating the availability of IGF-1 in joint and regulating cartilage matrix development, have been identified as targets of miR-140 [99,100,101].

### 4.2. tRNA-Derived Fragments in Knee OA

Mature tRNAs and nascent pre-tRNA transcripts are processed enzymatically to produce well-defined tRNA fragments (tRFs, reviewed in [108,109,110]). Sizes of tRFs range from 30 to 35 nt for tRNA halves and 14 to 26 nt for the shorter fragments. In higher eukariotes, tRNA halves are produced by the cleavage in the anticodon loop by an enzyme angiogenin, a member of the RNase A family [111]. tRFs are processed from the 5′ or the 3′ end of the mature tRNAs (5′-tRF and 3′-tRF) or immature tRNAs (pre-tRNA). The mechanisms of tRF formation are not yet fully understood, however, it was found that Dicer ribonuclease may be involved in the formation of tRF, despite the fact that the tRNA does not meet the classic substrate structural criteria for Dicer [112,113,114,115]. 

tRNA-derived fragments regulate gene expression on multiple levels, e.g., transcription, translation, ribosome biogenesis, stress granule formation, apoptosis, cell proliferation, retrotransposition, vesicle-mediated intercellular communication or intergenerational inheritance. Targeting the translational machinery is one of the well-understood mechanisms of their action [116,117,118,119,120] but there is a growing evidence of tRF loading on Argonaute proteins, whereby they direct the degradation of sequence matched targets [119]. Importantly, Dicer independence for the studied tRFs implicates yet more proteins in the RNA-induced silencing complex as tRF binding partners. Another clear difference of tRFs compared to the miRNAs is the fact that the former are not restricted to one domain of life, since they have been found in archaea, bacteria and eukarya. There are 2513 sequences of tRNA-derived fragments deposited in a tRFdb database, of which 552 correspond to human tRFs [120].

**Figure 3:**
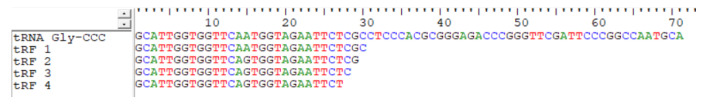
An example of identified tRFs derived from tRNA Gly-CCC. All tRFs were classified as 4 different tRFs.

Aberrant expression of tRFs was found in various human disease conditions, e.g., pathological stress injuries, multiple types of cancers and neurodegenerative diseases. Ageing is a leading risk factor that predisposes cartilage to pathological changes which may result in osteoarthritis [121]. In 2020, the first evidence that aged equine chondrocytes, compared to those of young ones, differentially express specific tRFs was published [122]. Eighty-one differentially expressed tRFs in young vs. old chondrocytes were discovered: 44 higher and 37 lower in old ones compared to the young ones. Moreover, in high grade OA compared to low-grade OA cartilage, the expression of eight tRFs was induced and three tRFs were reduced. A list of tRFs differentially expressed in osteoarthritis was filled by one more study. The human chondrocytes treated with IL-1β showed an increase in the expression levels of 14 tRFs and a decrease in 4 tRFs [123]. However, in our opinion the numbers of identified tRFs are overestimated, because in both studies tRFs derived from the same tRNA, differing only in length by one or two terminal nucleotides were identified as separate tRFs, as presented in Figure 3. This might be a false negative result from the sequencing, therefore the validation of the existence of stable tRFs in cells differing in one or two terminal nucleotides is of crucial importance. 

The summary of tRFs deregulated in OA is presented in Table 2. Additionally, we have compared the tendency of particular tRF up- or downregulation in OA with the codon usage. There exists a strong positive correlation between codon usage and tRNA content in organisms, and the extent of this correlation relates to the protein production levels of individual genes. We have observed that in most cases deregulated in OA, tRFs are derived from the tRNAs characterized by the anticodon complementary to the highly used codon. Interestingly, the same was observed in bacteria *Streptomyces coelicolor* [124] and *Trypanosoma cruzi* [125]. There are no reports so far concerning possible correlation between the expression of tRFs and the codon usage in humans.

Only one study so far aimed at discovering the function of tRFs in OA. Green et al., explored the role of the most upregulated 3’-tRF, derived from tRNA-Cys-GCA, in post-transcriptional gene regulation in IL-1β stimulated chondrocytes [123]. It appeared that this tRF posttranscriptionally regulates the Janus kinase 3 (JAK3) expression via AGO/RISC formation in chondrocytes. The JAK-STAT kinase pathway regulates the expression of many cytokines, including IL-6, which has an important role in the pathogenesis of multiple diseases, including osteoarthritis. 3′-tRF-Cys-GCA was able to suppress JAK3 kinase, which resulted in decreased expression of IL-6, indicating a potential role of this tRF in the regulation of OA pathogenesis.

## 5. Conclusions and Perspectives

Based on the functions of miRNAs in bone and cartilage and regulation in the development of knee osteoarthritis, miRNAs may provide an efficient cellular way for the regulation of gene expression during OA. The knowledge of tRNA-derived fragments in OA is still sparse. This is surprising taking into consideration that in multiple tissues and organisms, tRFs are as abundant as miRNAs. Therefore, a deeper understanding of the mechanism of expression and regulation of miRNAs and tRFs could be the key for OA diagnosis and prognosis.

Studies on miRNAs and tRFs have opened a new area of their potential use: in the diagnosis of OA as biomarkers. Extracellular miRNAs can be detected in almost all body tissues and fluids. The same is true for tRFs. There have been more than 100 clinical trials worldwide based on miRNA regulation to treat diseases, including cancers and cardiovascular conditions, but none for OA yet. The existing knowledge and understanding of small ncRNAs described in this review led us to the conclusion that there seems to be a lot more to come in the near future. The development of miRNAs-based diagnostic and therapeutic tools for human diseases, including OA, is definitely a long process. The increasing evidence of miRNAs and possibly tRFs’ involvement in OA signals is the beginning of this long process.

## Figures and Tables

**Table 1:** Summary of miRNAs which are differentially expressed in OA.

microRNA	Functional Effect of Increased/Decreased miRNA Expression	Ref.
miR-16 ↑	inhibition of FGFR by targeting SMAD3, altered proliferation and increased apoptosis, TGF-β signaling pathway affected	[71]
miR-21 ↑	inhibition of GDF-5 expression	[72]
miR-23a ↑	inhibition of FGFR by targeting SMAD3, altered proliferation and increased apoptosis, TGF-β signaling pathway affected	[71]
miR-25 ↑	WFA-induced upregulation of COX-2 expression, mediation of inflammatory responses in chondrocytes	[73]
miR-27b ↑	suppression of RC3H1 and QKI in OA synovium	[74]
miR-29b ↑	induction of chondrocyte apoptosis by targeting progranulin	[75]
miR-30b ↑	cartilage matrix degradation by targeting SOX9, ADAMTS-5 and ERG	[71]
miR-30c ↑	cartilage matrix degradation by targeting SOX9, ADAMTS-5 and ERG	[71]
miR-33a ↑	upregulation of MMP-13 and ECM degradation, regulation of cholesterol synthesis in TGF-β1/Akt/SREBP-2 pathway and cholesterol efflux-related ApoA1 and ABCA1	[76]
miR-34b ↑	modulation of OA chondrocyte proliferation by targeting CYR61, which inhibits ADAMTS-4 induced aggrecan degradation in cartilage	[73]
miR-104 ↑	unknown	[73]
miR-122a ↑	unknown	[73]
miR-126 ↑	unknown	[71]
miR-128a ↑	repression of chondrocyte autophagy by targeting Atg12	[77]
miR-130a ↑	sponging effect by aberrantly highly expressed HOTAIR lncRNA, massive apoptosis events, suppression of autophagy in chondrocytes	[78]
miR-133a ↑	inhibition of synovium pain-related genes, especially DST and TBXAS1	[79]
miR-135a ↑	unknown	[73]
miR-135b ↑	unknown	[73]
miR-139 ↑	inhibition of MCPIP1, activation of IL-6 and apoptosis	[73]
miR-144 ↑	unknown	[73]
miR-145 ↑	attenuation of TNF-α-driven cartilage matrix degradation by direct suppression of MKK4	[80]
miR-146a ↑	targeting SMAD4 disturbing TGF-β pathway, increased apoptosis, upregulation of VEGF	[81,82,83]
miR-147 ↑	unknown	[74]
miR-181a ↑	inhibition of chondrocytes proliferation and induction of apoptosis by targeting PTEN	[84]
miR-181b ↑	articular cartilage degeneration, destruction of lumbar facet joint cartilage	[85]
miR-184 ↑	unknown	[71]
miR-186 ↑	unknown	[71]
miR-200a ↑	unknown	[73]
miR-211 ↑	unknown	[73]
miR-215 ↑	inhibition of synovium pain-related genes, especially DST and TBXAS1	[86]
miR-218 ↑	inhibition the PI3K/Akt/mTOR signaling pathway, cartilage destruction	[87]
miR-224 ↑	inhibition of synovium pain-related genes, especially DST and TBXAS1	[86]
miR-299 ↑	unknown	[73]
miR-335 ↑	inhibition of synovium pain-related genes, especially DST and TBXAS1	[86]
miR-345 ↑	unknown	[71]
miR-365 ↑	upregulated expression of catabolic COL10A1 and MMP-13 by targeting HDAC4, activation of IL-6 and apoptosis	[88]
miR-381 ↑	upregulation of MMP13 and RUNX2 expression *via* targeting of HDAC4 cartilage degeneration	[89]
miR-455 ↑	regulating TGF-β signalling by suppression of the Smad2/3 pathway, targeting ACVR2B, SMAD2, CHRDL1	[90]
miR-486 ↑	inhibition of chondrocyte proliferation and migration by suppressing SMAD2 gene	[91]
miR-885 ↑	unknown	[71]
miR-9 ↓	increased chondrocytes proliferation and inhibition of cell apoptosis by targeting NF-κB	[92]
miR-26 ↓	induction of NF-κB signaling pathway	[93]
miR-27b ↓	inhibition of MMP-13	[94]
miR-29a ↓	inhibition of SMAD3, NFκB and WNT signaling pathway, part of ECM remodeling machinery	[71]
miR-107 ↓	unknown	[73]
miR-125b ↓	ADAMTS-4 - induced aggrecan degradation in cartilage	[95]
miR-127 ↓	decreased ECM synthesis by targeting IL-1β induced MMP-13	[96]
miR-140 ↓	targeting IGFR, ADAMTS5, MMP-13, IGFBP5 and RALA cartilage development and homeostasis, development of age-related OA-like changes	[97,98,99,100,101]
miR-148a ↓	targeting COL10A1, MMP13 and ADAMTS5, inhibition of hypertrophic differentiation, production and deposition of type II collagen and proteoglycan retention	[102]
miR-149 ↓	upregulation of TNFα, IL1β and IL6, activation of inflammation by targeting TNFα	[73,103]
miR-210 ↓	inhibition of NF-κB signaling pathway by targeting DR6 increasing inflammation	[104]
miR-221 ↓	activation of expression of catabolic genes, degeneration of cartilage tissues by upregulated expression of SDF1	[105]
miR-483 ↓	upregulation of catabolic genes expression, inhibition of TGF-β signaling pathway	[71]
miR-488 ↓	upregulation of catabolic genes expression by targeting ZIP8	[106]
miR-558 ↓	inhibition of COX-2 expression, inhibition of IL-1β-stimulated catabolic effect, altered inhibition of inflammatory factors	[107]

↑—upregulated, ↓—downregulated. FGFR—fibroblast growth factor receptor, SMAD3—mothers against decapentaplegic homolog 3, TGF-β—transforming growth factor β, GDF-5—growth differentiation factor 5, WFA—*Wisteria floribunda* lectin, COX-2—cyclooxygenase-2, RC3H1—ring finger and CCCH-type domains 1, QKI—Quaking, NFκB—nuclear factor kappa-light-chain-enhancer of activated B cells, SOX9—SRY-box transcription factor 9, ADAMTS-5—a disintegrin and metalloproteinase with thrombospondin type 1 motif 5, ERG—ETS transcription factor ERG, MMP-13—matrix metallopeptidase 13, ECM—extracellular matrix, CYR61—cysteine rich angiogenic inducer 61, Atg12—autophagy related 12, DST—dystonin, TBXAS1—thromboxane A synthase 1, MCPIP1—monocyte chemoattractant protein-induced protein 1, IL-6—interleukin 6, TNF-α—tumor necrosis factor alpha-like, MKK4—mitogen-activated protein kinase kinase 4, VEGF—vascular endothelial growth factor, PTEN—phosphatase and tensin homolog, COL10A1—collagen type X alpha 1 chain, HDAC4—histone deacetylase 4, RUNX2—RUNX family transcription factor 2; runt-related transcription factor 2, ACVR2B—activin A receptor type 2B, SMAD2—SMAD family member 2; small mothers against decapentaplegic homolog 2, CHRDL1—chordin like 1, RALA—Ras-related protein Ral-A, DR6—death receptor 6, SDF1—stromal cell-derived factor 1, ZIP8—zinc transporter 8,.

**Table 2:** tRFs which are deregulated in OA [122,123].

tRNA Isoacceptor	Codon Usage	5’-tRF	3’-tRF
tRNA-Ala-CGA	18.6		↑
tRNA-Ala-CGG	28.5		
tRNA-Ala-CGU	16.0		
tRNA-Ala-CGC	7.6		
tRNA-Arg-GCA	4.7		
tRNA-Arg-GCG	10.9		
tRNA-Arg-GCU	6.3		
tRNA-Arg-GCC	11.9		
tRNA-Arg-UCU	11.5		
tRNA-Arg-UCC	11.4		
tRNA-Asn-UUA	16.7		
**tRNA-Asn-UUG**	19.5		↑
tRNA-Asp-CUA	22.3		
**tRNA-Asp-CUG**	**26.0**	↑	
tRNA-Cys-ACA	9.9		
**tRNA-Cys-ACG**	12.2		↑
tRNA-Gln-CUU	29.0		
**tRNA-Gln-CUC**	**40.8**	↑	
tRNA-Glu-GUU	11.8	↓	↑
**tRNA-Glu-GUC**	**34.6**	↓	
tRNA-Gly-CCA	10.8		
**tRNA-Gly-CCG**	**22.8**	↓	↑
tRNA-Gly-CCU	16.3		
tRNA-Gly-CCC	16.4	↓	
tRNA-His-GUA	10.4		
**tRNA-His-GUG**	14.9	↑	
tRNA-Ile-UAA	15.7		
**tRNA-Ile-UAG**	**21.4**		↑
tRNA-Ile-UAU	7.1		
tRNA-Leu-AAU	7.2		
tRNA-Leu-AAC	12.6		
tRNA-Leu-GAA	12.8		
tRNA-Leu-GAG	19.4		
tRNA-Leu-GAU	6.9		
**tRNA-Leu-GAC**	**40.3**		↑
tRNA-Lys-UUU	24.0		↑
**tRNA-Lys-UUC**	**32.9**	↑	
tRNA-Met-UAC	22.3		
tRNA-Phe-AAA	16.9		
tRNA-Phe-AAG	20.4		
tRNA-Pro-GGA	17.3		
tRNA-Pro-GGG	20.0		
tRNA-Pro-GGU	16.7		
tRNA-Pro-GGC	7.0		↓
tRNA-Ser-AGA	14.6		
tRNA-Ser-AGG	17.4		
tRNA-Ser-AGU	11.7		
tRNA-Ser-AGC	4.5		
tRNA-Ser-UCA	11.9		
tRNA-Ser-UCG	19.4		
tRNA-Thr-UGA	12.8		
tRNA-Thr-UGG	19.2		
tRNA-Thr-UGU	14.8		
tRNA-Thr-UGC	6.2		
tRNA-Trp-ACC	12.8		
tRNA-Tyr-AUA	12.0		
**tRNA-Tyr-AUG**	**15.6**		↑
tRNA-Val-CAA	10.9	↓	
tRNA-Val-CAG	14.6		
tRNA-Val-CAU	7.0	↑	
**tRNA-Val-CAC**	**28.9**	↓	↑

Codon usage is presented in frequency/thousand. All human isodecoder tRNAs are presented. tRFs derived from the isodecoder tRNAs with the highest used codons are marked as bold. ↑—upregulated, ↓—downregulated.
